# Circulating Levels of Inorganic Pyrophosphate Influence the Risk and Clinical Outcomes in Calciphylaxis

**DOI:** 10.1016/j.jacadv.2026.103205

**Published:** 2026-09-03

**Authors:** Sagar U. Nigwekar, Api Chewcharat, Kevin O’Brien, Enock Arthur, Jennifer Howe, Lisa Laurion, Hervé Husson, Houda Bouchouari, Aaron Seto, Yves Sabbagh

**Affiliations:** a Division of Nephrology, Department of Medicine, Massachusetts General Hospital, Boston, Massachusetts, USA; b Research and Development, Inozyme Pharma Inc, Boston, Massachusetts, USA; c Frank H. Netter MD School of Medicine, Quinnipiac University, North Haven, Connecticut, USA; d Warren Alpert Medical School of Brown University, Providence, Rhode Island, USA

**Keywords:** calcific uremic arteriolopathy, dialysis, end-stage kidney disease, pain

**What is the clinical question being addressed?**

Although PPi is a critical inhibitor of vascular calcification, its role in calciphylaxis remains undefined.

**What is the main finding?**

Lower plasma PPi levels associate with higher risk, disease severity, and mortality in calciphylaxis, positioning PPi modulation as a strategy for future research.

Calciphylaxis is a rare, life-limiting syndrome defined by vascular calcification and neointimal proliferation.^1^ This pathological process induces microvascular occlusions within the subcutaneous and dermal adipose tissue, resulting in intensely painful, ischemic skin lesions. Primarily affecting patients with end-stage kidney disease (ESKD), this nongenetic condition carries substantial morbidity and mortality. Survival is often less than 1 year, with mortality largely driven by wound infections and cardiovascular events.^2^ The pathogenesis remains poorly understood and there are no approved therapies.

Animal models and studies of genetic mineralization disorders, including Generalized Arterial Calcification of Infancy, establish that a deficiency in the circulating calcification inhibitor inorganic pyrophosphate (PPi), drives vascular calcification and neointimal hyperplasia.^3^^,^^4^ However, the role of PPi in calciphylaxis remains unexplored. Given the lack of an animal model that recapitulates human calciphylaxis, we undertook the following studies approved by the Mass General Brigham Human Ethics Board.

1) Matched case-control study: Investigated the association between plasma PPi and calciphylaxis risk matching 30 cases with calciphylaxis to 30 controls on age (±10 years), sex, race, and ESKD status.
2) Prospective cohort study: Assessed the association of plasma PPi with disease severity and clinical outcome involving 70 patients with calciphylaxis followed over 12-weeks. This cohort included 30 cases of calciphylaxis who were analyzed in the aforementioned matched case-control study.

Patients were identified through the Partners Calciphylaxis Biobank and Patient Registry study (NCT03032835)—an active prospective registry of patients with confirmed diagnosis of calciphylaxis and their matched controls with detailed clinical and histopathological phenotyping and biosamples repository. Patients with calciphylaxis included in this study met our registry’s criteria for calciphylaxis which required a diagnosis made by 2 independent clinicians on review of clinical features (such as painful livedo reticularis, plaques or nodules, or necrotic lesions covered by eschar or full thickness open wound). In addition, skin histology review conducted by a dermatopathologist was supportive of the calciphylaxis diagnosis in all patients with calciphylaxis included in this study. Selection of cases for case-control study was dictated by the availability of matched controls. Fasting plasma PPi levels (predialysis for those who were treated with dialysis) were measured using an adenosine triphosphate sulfurylase-based luminescence assay. For case-control study, PPi levels were measured at enrollment. For cohort study, PPi levels were measured at enrollment and at week 6. We compared PPi levels between the case-control groups using Wilcoxon signed rank test and examined their association with calciphylaxis risk using adjusted conditional logistic regression. Furthermore, adjusted regression models assessed the relationship between plasma PPi and disease severity (skin lesion count and pain intensity), 6-week mortality, and 12-week mortality. The assumptions for the conditional logistic regression models included linearity of the log-odds, independence of observations, and the absence of multicollinearity. Continuous variables with non-normal distributions were log-transformed before inclusion in the linear regression analyses. The assumptions for the linear regression models included linearity, independence of observations, homoscedasticity, and the absence of multicollinearity.

All analyses were performed in SAS (version 9.4) with a *P* < 0.05 considered statistically significant.

At enrollment, for the entire calciphylaxis cohort (n = 70), the median age was 60 years (IQR: 53-70). Cohort was predominantly comprised of females (69%) and Caucasian race (71%). Comorbidities included ESKD (67%), diabetes (78%), coronary artery disease (44%) with a median body mass index (BMI) of 31 kg/m^2^ (IQR: 27-37), and 37% on warfarin. Serum calcium and phosphorous were mostly unremarkable. The median intact parathyroid hormone and alkaline phosphatase (ALP) were elevated (median intact parathyroid hormone: 130 pg/mL, IQR: 42-252; median ALP: 123 IU/L, IQR: 89-192) compared to reference ranges. The median skin lesion count was 3 (IQR: 2-4) and median pain intensity was 5 (IQR: 4-7) on a 0 to 10 scale. Mortality was 21% (15 dead, 55 alive) by 6-week and 27% (19 dead, 51 alive) by 12-week follow-up. Deaths were caused by either sepsis originating from wound infection or withdrawal from dialysis due to disease burden.

For case-control analysis, patient characteristics were well-balanced between cases (n = 30) and matched controls (n = 30), except for serum ALP which was higher in cases (median: 132 IU/L; IQR: 102-214) than in controls (median: 99 IU/L; IQR: 75-147, *P* = 0.03). ESKD was present in 67% of cases and controls (Table 1). PPi levels were significantly lower among patients with calciphylaxis compared to matched controls, (cases: median: 248 nM, [IQR: 110-323]; controls: median: 611 nM, [IQR: 361-799]; *P* < 0.001). The finding of lower PPi levels in calciphylaxis compared to controls was consistent in analyses stratified by presence or absence of ESKD. Moreover, PPi levels in patients with calciphylaxis were substantially lower than those previously reported for healthy adults by Khursigara et al^5^ (median: 2,167 nM [IQR: 1,787-2,969]). In conditional logistic regression model adjusted for ALP, phosphate, and BMI, each 100 nM reduction in PPi was associated with a 68% higher odds of calciphylaxis (OR: 1.68, 95% CI: 1.26-2.42).

**Table 1:** Baseline Characteristics Between Calciphylaxis Cases (n = 30) and Controls (n = 30)

	Cases (n = 30)	Controls (n = 30)	*P* Value
Age, years, median [IQR]	58 [50-68]	63 [55-72]	0.18
Female, n (%)	20 (67%)	20 (67%)	NA
White, n (%)	26 (87%)	26 (87%)	NA
BMI, kg/m^2^, median [IQR]	30.5 [26.8-36.5]	29.7 [23.8-38.7]	0.32
ESKD (%)	20 (67%)	20 (67%)	NA
Diabetes mellitus, n (%)	21 (70%)	18 (60%)	0.42
Peripheral artery disease, n (%)	13 (43%)	4 (13%)	0.001
Warfarin use, n (%)	9 (30%)	6 (20%)	0.37
Serum calcium, mg/dL, median [IQR]	9.2 [8.6-9.7]	9.3 [8.4-9.6]	0.58
Serum phosphate, mg/dL, median [IQR]	4.3 [3.5-5.4]	4.3 [3.6-4.9]	0.59
Serum ALP, IU/L, median [IQR]	132 [102-214]	99 [75-147]	0.03
Serum iPTH, pg/mL, median [IQR]	94 [47-205]	152 [65-436]	0.14
Serum 25-hydroxyvitamin D, ng/mL, median [IQR]	21.5 [13.5-36.7]	21.0 [16.0-35.0]	0.26
Baseline PPi, nM, median [IQR]	248 [110-323]	611 [361-799]	<0.001
Skin lesion count, median [IQR]	4 [2-6]	Not applicable	Not applicable

ALP = alkaline phosphatase; BMI =body mass index; ESKD = end stage kidney disease; iPTH = intact parathyroid hormone; PPi = inorganic pyrophosphate.

At enrollment, PPi levels negatively correlated with the number of skin lesions (r_s_ = −0.26; *P* = 0.03) but not with pain intensity. Compared to calciphylaxis patients who were alive by 6 weeks, those who died had significantly lower enrollment PPi levels (245 nM [IQR: 78-543] vs 727 nM [IQR: 341-1,232], *P* = 0.002). Among ESKD patients with calciphylaxis, each 100 nM reduction in enrollment PPi was associated with ∼50% higher 6-week mortality after adjusting for age, sex, and BMI (OR: 1.54; 95% CI: 1.09-2.18). A similar association was also noted between enrollment PPi and 12-week mortality. There was a substantial within-individual variability between 6-week and enrollment PPi levels.

In conclusion, our findings—despite limitations in sample size and observational design—position circulating PPi as a dynamic marker for the risk, severity, and mortality of calciphylaxis. Given that trials for therapies aimed at increasing or preserving PPi are already underway for genetic mineralization disorders,^4^ we propose that these approaches that modulate PPi levels merit investigation to improve outcomes attributed to calciphylaxis.

## Funding support and author disclosures

This collaborative study was funded by Inozyme Pharma Inc. Dr Nigwekar has been supported by the American Heart Association’s National Center for Research Program Winter Fellow-to-Faculty Transition Award (15FTF25980003), the KL2/Catalyst Medical Research Investigator Training award (an appointed KL2 award) from Harvard Catalyst, the Harvard Clinical and Translational Science Center (National Center for Research Resources and the National Center for Advancing Translational Sciences, National Institutes of Health Award KL2 TR001100), the National Kidney Foundation’s Young Investigator Award, the Fund for Medical Discovery Award from Massachusetts General Hospital’s Executive Committee on Research (R00000000007190), and the American Heart Association’s National Center for Research Program Summer Mentored Clinical and Population Research Award (15CRP22900008) to set up the Partners Calciphylaxis Biorepository and Patient Registry (NCT03032835) through which patient enrollment for the studies presented here was conducted. Dr Chewcharat has received grant support from American Kidney Fund Clinical Scientist in Nephrology Award. Dr Nigwekar received compensation from the American Society of Nephrology for his role as the Deputy Editor of the Clinical Journal of Society of Nephrology and from the National Kidney Foundation for his role as Associate Editor of the Advances in Kidney Disease and Health; and consulting fees and/or funding from CSL Behring, Fresenius Medical Care, Inozyme Pharma Inc, Epizon Pharma, Vera Therapeutics, Alexion, RubiconMD, and Sanofi; and royalty payments from UpToDate. Drs O’Brien, Howe, Laurion, Husson, and Sabbagh were employees of Inozyme Pharma Inc. All other authors have reported that they have no relationships relevant to the contents of this paper to disclose. The content is solely the responsibility of the authors and does not represent the official views of Harvard Catalyst, Harvard University and its affiliated academic health care centers, the National Institutes of Health, or the United States Department of Agriculture.
